# Assessing knowledge, attitudes, and practices and demand-side interventions for combating substandard and falsified medicines: a scoping review

**DOI:** 10.1080/20523211.2025.2550369

**Published:** 2025-09-12

**Authors:** Eishita Pal, Lubna Merchant, Alain K. Koffi, Reema Mehta, Jean Christophe Rusatira, Lev Kubiak, Henry Joseph Michtalik, Patrick Caubel, Saifuddin Ahmed

**Affiliations:** a Department of Population, Family and Reproductive Health, Johns Hopkins Bloomberg School of Public Health, Baltimore, MD, USA; b Worldwide Medical & Safety, Pfizer, New York, NY, USA; c Department of International Health, Johns Hopkins Bloomberg School of Public Health, Baltimore, MD, USA; d Johns Hopkins School of Medicine, Baltimore, MD, United States; e Global Security, Pfizer, New York, NY, USA

**Keywords:** Substandard and falsified medicines, counterfeit medicines, fake medicines, drug safety

## Abstract

**Background:**

The proliferation of substandard and falsified medical products (SFM) poses a significant threat to public health globally. Despite rigorous surveillance and law enforcement efforts, risk of exposure to SFM is on the rise, notably through online pharmacies. The current interventions predominantly target the pharmaceutical supply chains through legal and regulatory frameworks, while there is a noticeable deficiency in focusing on interventions for healthcare providers and consumers. This scoping review aims to summarise the current literature on SFM, focusing on their health and economic consequences, and to assess the knowledge, attitudes, and practices of healthcare providers and the general public.

**Methods:**

A comprehensive literature search was conducted across PubMed, Embase, and Scopus databases, focusing on studies from the past 15 years that provided estimates on mortality, morbidities and economic impacts of SFM and covered the following topic areas: knowledge, attitudes and practices of healthcare providers, patients and general public; and population level interventions regarding SFM. We exclude non-peer-reviewed literature.

**Results:**

A total of 78 studies met the inclusion criteria and were analyzed. These studies suggest that the data on adverse effects on health and economic impact of SF medicines are predominantly based on statistical models, and empirical data are grossly lacking. Knowledge of risks, identification of SFM, and reporting to regulatory authorities are substantially low among healthcare providers and general public.

**Discussion:**

This review highlights the need for innovative, targeted strategies – such as digital health interventions, enhanced training programs for healthcare providers, and context-specific public awareness campaigns – to bridge the gap between awareness and effective practice.

**Conclusions:**

Our study underscores that a multifaceted approach must not only reinforce regulatory frameworks and surveillance systems for protecting the supply chains but also proactively empower both health providers and consumers to identify and combat SFM in today’s rapidly evolving digital landscape.

## Background

The proliferation of substandard and falsified medicines (SFM) in the last few decades represents a complex and widespread challenge to global public health. The World Health Organization (WHO) identified this problem as ‘one of the urgent health challenges for the next decade’ (World Health Organization, 2020). The WHO defines substandard medicines as ‘authorised products that fail to meet either their quality standards or their specifications, or both’ and falsified medical products are those that ‘deliberately/fraudulently misrepresent their identity, composition or source’ (World Health Organization, 2017b). These drugs are likely to be seriously toxic due to overdose or contamination with toxic substances and completely ineffective due to insufficient or no active ingredients. Their usage leads to deaths and treatment failures and increases the risks of complications and other morbidities. Extensive usage of SF antimicrobial and antimalarial drugs is considered a major attributable factor for increasing drug resistance to malaria, tuberculosis (TB), and other infectious diseases in low- and middle-income countries(LMICs) (Hoellein et al., 2022; Zabala et al., 2022). Their ineffectiveness leads to erosion in public trust in the use of drugs and vaccines (Amankwah-Amoah, 2022; Ozawa & Stack, 2013; Ware et al., 2023). Concurrently, SF drugs impose significant economic burdens on patients and health systems due to the costs associated with ineffective treatments and the management of complications and prolonged illnesses (Ozawa et al., 2022).

The WHO estimates that about 10.5% of drugs in low- and middle-income countries are likely to be SFM (World Health Organization, 2017a). A systematic review of 25 countries found that 11–48% (median 28.5%) of drugs were substandard or SF (Almuzaini et al., 2013). Economic analysis suggests that the market value of SF drugs is between $200 billion and $431 billion annually, and the US Industry alone is annually losing between $37.6 billion and $162.1 billion in revenues (Miller & Winegarden, 2020).

No country is immune; SF drugs have been found on every continent and in almost every country, ranging from Australia (Hensey & Gwee, 2016) to Guinea-Bissau and Nigeria (Otte et al., 2015), to Panama (Bogdanich & Hooker, 2007). This problem also persists in high-income countries with strong regulatory enforcement (Mackey & Liang, 2011).

The danger of SFM in the global supply chain was first officially recognised as a public health threat in 1985 during the WHO’s Conference of Experts on Rational Drug Use. Since then, awareness and initiatives have grown and the WHO, public health officials, drug regulatory organisations, law enforcement agencies, and pharmaceutical industries have been working together to combat the relentless threat of SFM. The European Union (EU) introduced harmonised safety and control measures across EU countries through the Falsified Medicines Directives (FMD) in 2011 for the verification and identification of falsified medicines at EU borders and within the EU countries. The US Congress passed the Drug Supply Chain Security Act (DSCSA) in 2013 to enhance unit-level tracing of prescription drugs throughout the supply chain. India implemented a track and trace system, Drug Authentication and Verification Application (DAVA), in 2015 for tracing the movement of authenticated pharmaceutical products. Better detection technology and strong vigilance have also contributed to detecting higher trends of pharmaceutical crime incidents. Nevertheless, although pharmaceutical industries, government agencies, and international organisations have undertaken numerous strategies over the last few decades to combat the threats of SFM, the spread of counterfeit drugs has not decreased but increased at an alarming level. This is particularly evident in the context of online pharmacies, where the Internet is increasingly exploited to facilitate the global trade in counterfeit pharmaceuticals (Lavorgna, 2015). The rapid growth of the counterfeiting drugs market, documented evidence of thousands of deaths, and the threat of counterfeiting lifesaving medicines, including COVID-19 drugs and vaccines, further exaggerate the urgency to combat the threat (Srivastava, 2021; Tesfaye et al., 2020; World Health Organization, 2021b; Ziavrou et al., 2022).

The WHO has identified several key drivers contributing to the global spread of SF medicines, including weak regulatory systems with insufficient oversight and enforcement, complex supply chains that increase the risk of product tampering, limited access to affordable medicines driving consumers toward unregulated sources, lack of public awareness about SF medicine risks, and corruption within regulatory and enforcement bodies. A number of review studies have examined the studies on the methods of detecting SFM in supply chains (Ciapponi et al., 2021; Krakowska et al., 2016; Kumar & Agrawal, 2014), the studies on the prevalence of such drugs in the market (McManus & Naughton, 2020), and studies on the interventions relating to law enforcement and regulatory strengthening (Martins et al., 2022). A systematic review suggests low-quality evidence that regulatory measures may be effective in reducing the prevalence of counterfeit and substandard drugs (El-Jardali et al., 2015). There are significant concerns that studies on SF drugs are predominantly focused on supply chain threats and limited information is available on the demand-side factors (Ofori-Parku, 2022). Most strategies to combat these problems are directed at the stakeholder levels and emphasise supply chain processes, trace and track technological interventions, and legal mechanisms. However, current interventions pay less attention to consumer judgment and decision-making aspects (Ofori-Parku & Park, 2022), especially the attitudes and behaviours of consumers and healthcare providers towards counterfeit medicines (Bird, 2007).

It is often considered that patients, consumers, and healthcare providers lack a comprehensive understanding of the risks associated with SF and illicit medicines. Although awareness of these issues is increasing, recent evidence indicates that substantial gaps persist in translating knowledge into effective counselling and practice. For example, a recent cross-sectional study in Ethiopia found that while many healthcare providers were aware of the dangers posed by SF medicines, only a small proportion felt adequately prepared to counsel patients on these risks.^87^ Similarly, surveys among pharmacists in Saudi Arabia have reported deficiencies in both training and confidence regarding the identification and reporting of SF medicines.

To address this challenge, this scoping review aims to examine the current landscape of SFM, specifically to examine the public health aspects of the problems and demand-related public health interventions, such as improving knowledge, access, and affordability to safe drugs. Many papers we included in this review used the term counterfeit drugs (Isles, 2017), instead of *substandard and falsified*, and it was difficult to ascertain whether these studies examined or included substandard drugs; consequently, we used the term counterfeit in such contexts. The panel explains briefly the concerns of intellectual property issues while using the term counterfeit medicines.

## Methods

### Data and methods

We have adopted the five-stage approach proposed by Arksey and O’Malley (Arksey & O’Malley, 2005) for conducting this scoping review. We followed the Preferred Reporting Items for Systematic Reviews and Meta-Analyses extension for Scoping Reviews (PRISMA-ScR) checklist for presenting the results (Tricco et al., 2018).

### Database sources and search strategy

A comprehensive literature search on SF medicines, their detrimental effects, and intervention strategies for their mitigation was conducted using: PubMed, Embase, and Scopus. To ensure a high level of specificity and maintain manageability of the search results, we focused on a set of limited keywords. We specifically used the following search terms in the title and abstract fields:

1. PubMed (*n* = 1132 with English language as a filter)
  **‘**counterfeit drug*’ OR ‘counterfeit medicine*’ OR ‘falsified drug*’ OR ‘falsified medicines*’ OR ‘fake drug*’ OR ‘fake medicine*’ OR ‘substandard medicine*’ OR ‘substandard drugs*’
2. Embase (*n* = 745)
  **‘**counterfeit drug*’:ti OR ‘counterfeit medi*’:ti OR ‘falsified med*’:ti OR ‘substandard med*’:ti OR ‘falsified drug*’:ti OR ‘substandard drug*’:ti OR ‘fake med*’:ti OR ‘fake drug*’:ti
3. Scopus (*n* = 1095)
  ((counterfeit* W/3 drug*) OR (counterfeit* W/3 medicine*) OR (fals* W/3 drug*) OR (fake* W/3 drug*) OR (fals* W/3 medicine*)) AND NOT (review)

We acknowledge that the choice to focus solely on these specific terms may result in potential omissions. Broader search terms such as ‘quality [of medicines]’, ‘medical products’, ‘health products’, ‘vaccines’, or phrases related to ‘knowledge, attitudes, and practice’ were not included, as preliminary tests indicated that their inclusion generated a large number of irrelevant hits. These decisions were made to enhance the quality and reliability of our findings by ensuring that only rigorously evaluated, high-quality evidence from peer-reviewed sources was included.

### Inclusion and exclusion criteria

This review broadly covered two thematic areas: public health impact of SFM in terms of health and economic threats, and knowledge, attitudes and practices regarding SFM. To be included in the review, studies had to meet the following criteria: (i) original research published in peer-reviewed journals, (ii) publications during the last 15 years, approximately between 2007 and early-2024 that provided analytical estimates on mortality, morbidities and economic impacts of SFM and covered the following topic areas: knowledge, attitudes and practices of healthcare providers (physicians, nurses, pharmacist) and consumers (patients and general public) regarding SFM and population level interventions. Excluded were conference abstracts published in journals, and studies with a primary focus on purchasing illicit drugs. Additionally, gray literature such as dissertations and theses, conference presentations, review papers, reports, newspaper articles, letters to the editor, editorials, and commentaries was excluded.

### Role of the funding source

The funder of the study has no role in study design, search strategy, study selection, and determining the scopes and preparation of the manuscript.

### Literature search results and data extraction summary

**Figure 1.**
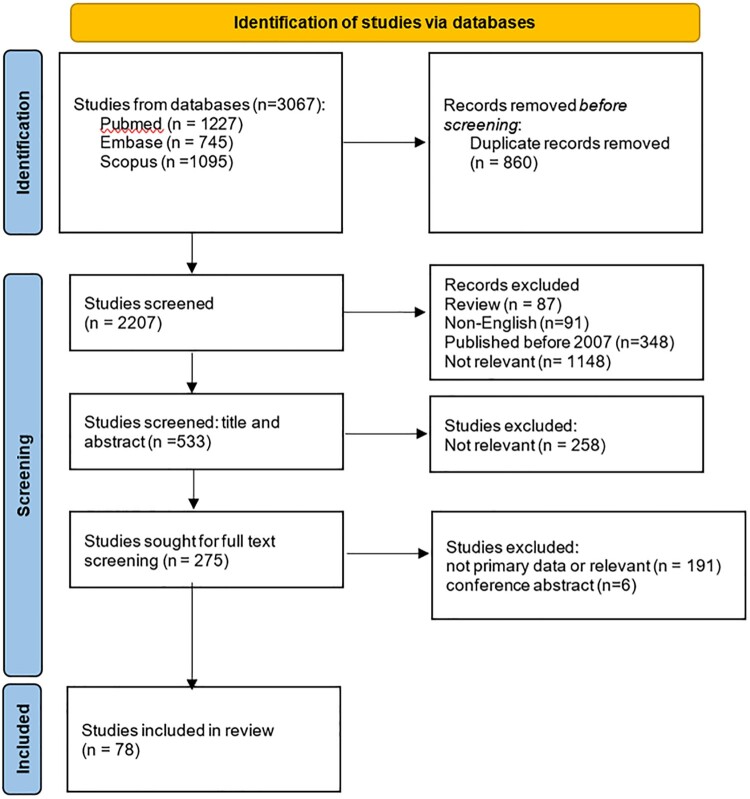
PRISMA-ScR flow chart showing search strategy and study selection process.

A comprehensive search across three databases generated a total of 3,067 results. We identified 1,227 studies from PubMed, 745 studies from Embase, and 1,095 studies from Scopus databases. Of these records, 860 were duplicates and they were excluded. The selection process of the studies is shown in Figure 1. A rigorous screening process involving title and abstract evaluation (*n* = 533) followed by a thorough full-text review (*n* = 275) was conducted, adhering to predetermined inclusion criteria. Ultimately, 78 studies were deemed eligible and included in the scoping review (shown in Supplemental Material with the authors’ list, study design, study countries, sample size, study population, and key selected results).

## Results

### Pervasive health and economic threats of SFM

**Figure 2.**
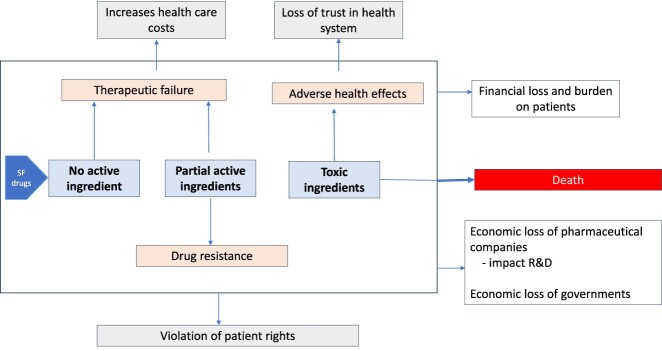
Adverse public health and economic consequences of substandard and falsified medicines.

From the public health perspective, the main consequential features of SFM are that they may contain no or partial active pharmaceutical ingredients (API) and may contain toxic materials adversely affecting health and health system enormously with profound economic consequences (Figure 2). Although the adverse health impacts of SFM are catastrophic, measuring the overall public health burden, including the number of deaths, the incidence of complications, and economic loss due to SF drugs is challenging. The reporting of deaths from SFM is still very poor and capturing their attributable effects is complex.

A very limited number of studies examined the public health impacts with empirical data. We identified 13 published papers in the reference period that examined or estimated the public health impacts of SFM (Supplemental Material Table S1). An analysis of published studies on health consequences of falsified medicines between 1972 and 2017 found only 48 incidence reports and estimated approximately 7,200 casualities with 3,604 deaths globally (Rahman et al., 2018), which is likely grossly underestimated.

Most estimates of the mortality effects of SFM at a population level are predominantly derived from statistical modeling. Model-based analysis by the WHO/University of Edinburgh suggests there could be 8,688 to 72,430 excess deaths of children from the ineffective treatment of severe pneumonia globally (World Health Organization, 2017a). Another analysis by the WHO/London School of Hygiene and Tropical Medicine suggests that the substandard and falsified antimalarial drugs contributed to an additional 72,000–267,000 deaths annually in sub-Saharan Africa (SSA).

Six studies applied agent-based modeling, predominantly the Substandard and Falsified Antimalarial Research Impact (SAFARI) model, and conducted simulations to estimate the impact of antimalarial drugs on under-five deaths due to malaria in a number of sub-Saharan African countries (Beargie et al., 2019; Evans et al., 2019; K. D. Jackson et al., 2020; Ozawa, Evans, et al., 2019; Ozawa, Haynie, et al., 2019; Renschler et al., 2015). These studies suggest about 3.75% (Renschler et al., 2015) to 8.2% (K. D. Jackson et al., 2020) of excess deaths due to SFM. The poorest populations were disproportionately affected by SFM: the number of deaths among the poorest wealth quintile due to substandard and falsified antimalarials was 12.7 times higher than that of the wealthiest quintile (Evans et al., 2019). A similar model-based analysis of the impact of substandard uterotonic drugs like oxytocin to prevent postpartum hemorrhage, the leading cause of maternal mortality globally, contributes to $18.8 million in economic loss and 100 maternal deaths annually in Ghana alone (Bautista et al., 2024).

Other data sources on deaths and catastrophic health effects by SFM are from case studies, case–control studies, and investigative procedures following an unusual outbreak of a health condition (Kao et al., 2009; Peyraud et al., 2017; Rentz et al., 2008; Vallersnes et al., 2009). An examination of the suspected outbreak of meningitis in the Democratic Republic of Congo found dystonia among 1,029 patients was due to falsified diazepam containing haloperidol, which caused 11 deaths (Ahmed et al., 2022). Matched case–control study design was used in Panama to identify cough syrup with diethylene glycol as the cause of acute renal failure (Rentz et al., 2008). In Canada, several deaths were attributed to counterfeit Norvasc, a drug for treating hypertension and angina, which contained only talcum powder (The Partnership For Safe Medicines, https://www.safemedicines.org/2005/09/4-deaths-tied-to-counterfeit-drugs-dispensed-at-ontario-canada-pharmacy.html, 2005). Counterfeit phosphodiesterase type-5 inhibitors, (PDE5i, e.g. Viagra) one of the most common counterfeit drugs available on online Internet market, pose immense direct and indirect health and economic risks to users (G. Jackson et al., 2010; G. Jackson et al., 2012; Sugita & Miyakawa, 2010).

Life-saving drugs, including vaccines, are not immune from counterfeiting. The deaths of 2,500 people in Niger were attributed to the use of vaccines lacking any active ingredient (Burns, 2006). Counterfeit COVID vaccines, specifically Covishield, were detected in India (Choudhary et al., 2021; World Health Organization, 2021a). Additionally, counterfeit COVID-19 vaccines have also been found in South Africa (Aborode et al., 2022), Mexico, and Poland (Amankwah-Amoah, 2022).

Illicitly manufactured Fentanyl drugs were involved in 41.4% of deaths from overdose in the USA, which were mostly counterfeit pills (O'Donnell et al., 2021). Evidence of counterfeiting was present in 24.5% of overdose deaths among adolescents aged 10–19 years (Tanz et al., 2022). The CDC data from 34 states and Washington, DC show that the drug overdose-related deaths from counterfeit pill use more than doubled from 2% in 2019 to 4.7% in 2021 (O'Donnell et al., 2023). The California Poison Control System in San Francisco division identified 8 patients who experienced adverse effects associated with the ingestion of counterfeit alprazolam tablets found to contain fentanyl and, in some cases, etizolam (Arens et al., 2016). Owing to increasing demand, counterfeit benzodiazepine tablets with fentanyl are currently available at an alarming rate (Tobias et al., 2021). In 49 states of the USA, the presence of fentanyl was found in confiscated pills with deaths attributed to use in 38 states (Palamar et al., 2022). Counterfeit versions of Xanax tablets obtained from the unregulated drug market, were found not to contain alprazolam but contained fentanyl (Arens et al., 2016; Tobias et al., 2021).

### Knowledge, attitudes, and practices of healthcare providers and consumers regarding SFM

We have identified 55 publications that examined the knowledge, attitudes, and practices of healthcare providers and the general public, including patients (Supplemental Material Table S2). We summarise the key findings below.

#### Knowledge about SFM and perceived burden

Healthcare providers’ knowledge and attitudes towards SF drugs significantly influence their handling and reporting practices. Overall, our review suggests that knowledge of SFM among healthcare providers is low in many settings, especially in high-income countries. A study of community pharmacists in Italy suggests that about 7% of the respondents had previous experience with SF drugs but almost one-third of the respondents didn’t know about SF drugs (Lombardo et al., 2019). In Sweden, 80% of the community pharmacists knew the definition of SF drugs; however, 74% of them could not recognise the common European logo for authorised online pharmacies (Persson et al., 2022). Among physicians In Sweden, about 78.5% heard about illegal and falsified medicines but expressed a need to have more knowledge (Funestrand et al., 2019). When asked in an open-ended question how they would act if they had a strong suspicion their patient was taking SFM, 64.5% answered that they would try to inform the patient about the risk but only 17.5% answered to advise the patient against using the drug; only 5% physicians mentioned the possibility of reporting to regulatory authority. In Ukraine, only 11.4% of doctors have an awareness of counterfeit medicines, compared to 42.3% among pharmacy specialists, 59.7% among State Medical service experts, and 73.5% among consumers (Lebed & Nemchenko, 2021). A survey of pharmacists in California, USA, found that 12% of the respondents encountered counterfeit medicines but only 59.3% considered counterfeit medicines to pose a problem (Law & Youmans, 2010, 2011).

Low knowledge of SFM among pharmacists was also found in Iran; only 11.6% could correctly identify the examples of drugs that were likely to be counterfeit (Shahverdi et al., 2012). In Lebanon, pharmacists often identify SFM by their lack of effectiveness and low costs (Sholy et al., 2018). In India, knowledge about counterfeit drugs showed that only 22% of the healthcare providers knew about counterfeit drugs and 36% per cent of them could not distinguish between genuine and fake drugs (Nagaraj et al., 2015). Low knowledge of the correct meaning of counterfeit drugs was also found among registered doctors in India (Yadav et al., 2018). About one-third (30.7%) of the pharmacist in a survey in Nigeria acknowledged that their current knowledge and skills were inadequate to detect counterfeit medicines (Adigwe et al., 2022). Lack of knowledge was also found among pharmacists in Saudi Arabia regarding the technology available to detect SFM and the regulatory program – the ‘Drug Track and Trace System’ – implemented to curb SFM (Ali & Barrett, 2023). A survey of pharmacists in Egypt suggests substantial misperception about counterfeit medicines – about two-thirds of the respondents perceived them as inactive, 61.7% as harmful, and 28.6% as less effective or inexpensive (Bashir et al., 2020). About half of the pharmacists were aware of medicine authentication techniques but most respondents (88.0%) were not aware of the law or legislation to control or reduce counterfeit medicines. A study in Nepal found that pharmacists, paramedics, and nurses had lower scores in knowledge of counterfeit medicines compared to physicians (Chaudhary, 2023). A study in Eritrea suggests that most pharmacists could recognise the differences between falsified and substandard drugs (Fitsum et al., 2023). On inquiring about the problems of SF drugs, most responded with adverse effects (82.5%0 and treatment failure (66.2%). About 63% of these pharmacists suggested they encountered SFM. In Nigeria, 95% of the healthcare providers in a survey perceived that SFM are higher than 10% in the country and almost 42% considered that the prevalence is likely more than 50% (Joda et al., 2017).

Several studies attempted to measure consumer behaviour toward counterfeit drugs, including with a valid tool (Alfadl et al., 2013). A comparison of knowledge between pharmacists and the public in Qatar suggests no differences in the way they perceive counterfeit medicines (Alfadl et al., 2018). Often, consumers recognise that the purchased drugs are likely SFM when they find out the drug is ineffective and upon closer observation show differences in the labels compared to previous purchase (Ekoh et al., 2022). A study in Sudan suggests consumers often use the side effects as the main characteristic to identify the CFM (Wagiealla et al., 2022). An online survey conducted across Europe, Asia, Africa, America, and the Middle East found that about 37.5% of the respondents considered counterfeit medicines as a national health problem, where only 30.6% (95% CI: 25.6%–35.7%) of the respondents could correctly identify counterfeit drugs based on the suggested characteristics (El-Dahiyat et al., 2021). An examination of public awareness in Tanzania found that about half (44.4%) of the respondents could not distinguish between genuine and counterfeit medicines (Mhando et al., 2016). Most respondents (45.7%) opined that the use of counterfeit drugs could be reduced through sensitising people’s awareness. Social media and the pharmacy were identified by the consumers in Sudan as their main sources of awareness about SFM (Wagiealla et al., 2022).

In-depth interviews of key stakeholders in medicine regulation and law enforcement in South Africa suggested that law enforcement is often suboptimal because of poor engagement and information sharing among stakeholders. They also opined inadequate legislation, lack of institutional/organisational capacity, lack of enforcement and market control, lack of stakeholder collaboration and information sharing, and low education and awareness are the obstacles to combating counterfeit medicines (Moshoeshoe et al., 2022). About 13% of the managing executives of wholesalers of pharmaceutical products in Cambodia have reported that they encountered counterfeit medicines; however, their definitions and perceptions of counterfeit vary widely (Khan et al., 2011).

Knowledge often does not translate into good practice. In Malaysia, a survey suggests that about half of the respondents had a moderate level of knowledge but over 50% of respondents demonstrated poor practice against identifying counterfeit medicines (Por et al., 2020). Large proportion of (42.1%) respondents also had mixed opinions about the harmful effects of these SFM. Despite universal knowledge about the methods of identifying SFM, physicians in Nigeria were not utilising SFM checking methods at the point-of-care due to time constraints (Iloh et al., 2021). A survey of the public in Lebanon shows that the vast majority (93.4%) of respondents had heard about counterfeit medicines but almost half (48.5%) did not know or answer the question on how to compare counterfeit to original medicines (Sholy & Saliba, 2018). Similar patterns of findings were also observed among healthcare providers in Ethiopia (Siraj et al., 2022).

#### Perceptions about interventions to prevent SFM in supply chain*s*

Opinions about effective interventions vary across countries. Pharmacists in Nigeria in a survey identified inadequate enforcement of law (93.8%), high costs of pharmaceuticals (77.4%), inadequate public awareness (93.5%), the existence of unregulated open drug markets (93.7%) are the major reasons for CFM drugs in the country (Obi Peter Adigwe, 2023). The vast majority (92.9%) of them opined that the use of drug testing and screening technologies could help to improve the detection of fake drugs in the country. A survey of the National Medicines Regulatory Authorities (NMRAs) of the 16 Member States within the Southern African Development Community (SADC) region in 2018–2019 examined the implementation plan of the WHO’s prevention, detection and response (PDR) program in countries to protect the impact of substandard and falsified products. Of the respondents who participated in the survey, 91.7% (11 of 12) reported that they regularly receive the WHO alerts for SF medical products but 75% (9 of 12) of them responded that that the country has no policy framework to support the PDR program (Kniazkov et al., 2020).

#### Training needs of healthcare providers

About three-quarters (72.3%) of the study participants in a survey of pharmacists in Nigeria indicated that regular training for pharmacists is necessary to enhance the capacity to detect counterfeit pharmaceuticals (Adigwe, 2023). In UK, pharmacists favourably opined training courses can improve pharmacist’s knowledge (Barrett & Al-Mousawi, 2018). A study in Eritrea suggested that only 16.5% of the pharmacists received any training on falsified medical products (Fitsum et al., 2023). Formal educational training in academic years has been shown to improve knowledge and competencies in detecting SFM. An evaluation study that examined the effects of dedicated educational courses for undergraduate pharmacy students has found increased overall students’ scores on SFM knowledge after receiving the training in Cameroon, Senegal, and Tanzania (Kusynova et al., 2023). However, very few educational institutions offer formal educational courses on SFM. A study examined the availability of dedicated SFM education in 37 countries and found that 33% of the universities did not teach SF medical product issues to pharmacy students (Kusynová et al., 2021).

#### Counselling of patients by healthcare providers

Although counselling of patients has been discussed and advocated by the WHO and regulatory agencies, very few studies have examined the HCP’s role in counselling patients on the dangers of SFM and the risks of purchasing drugs from unauthorised sources (Shrivastava et al., 2014).

A study of pharmacists in the USA in 2021 suggested that 58% lacked confidence in their ability to counsel patients on the identification of illegal online websites and 75% of them were unfamiliar with resources available to help consumers to identify authorised legitimate online pharmacies. About 6% of the pharmacists reported knowledge of patient’s adverse experiences with drugs obtained from online pharmacies (Hertig et al., 2021). Another study in California, USA, suggests that 56.5% of pharmacists never discuss the issue of falsified medicines with patients (Law & Youmans, 2011). In Poland, a survey of healthcare providers shows that about 56.2% of physicians and 50.7% of nurses (56.2% vs. 50.7%) usually do not warn patients against counterfeit medicines. About 71.2% of the layperson respondents reported that they had never been informed by physicians or nurses against purchasing medicine from unknown sources and the risks of obtaining drugs from online sources (Binkowska-Bury et al., 2013).

#### Reporting suspicious SFM to a regulatory authority

Many countries have instituted reporting of suspicious pharmaceutical products to regulatory authorities but our review suggests that these reporting systems are often not used by healthcare providers. A study of pharmacists in Saudia Arabia found that about half of the respondents never used the Saudi Vigilance services for reporting falsified medicines and did not know any technologies available to detect falsified medicines (Ali & Barrett, 2023). In Eritrea, the standard form used to report suspected falsified medical products could be recognised by only 13.8% of the pharmacists; not knowing how to report to the Eritrean Pharmacovigilance System at the National Medicines and Food Administration was the most cited (55.6%) barrier to report SFM (Fitsum et al., 2023). About 72% of the Swedish community pharmacists expressed the need for additional knowledge about SF drugs (Persson et al., 2022). Although the nurses and physicians have a high level of awareness in some settings, most healthcare providers lack the knowledge to report suspected SF drugs and counsel the patients against the drugs from unknown sources (Binkowska-Bury et al., 2012; Binkowska-Bury et al., 2013). Difficulties in identifying SFM, the complexity of using the existing reporting system, and fear of reputational repercussion were reported as the key barriers to reporting suspected SFM by the healthcare providers in Tanzania and Indonesia (Wagnild et al., 2023). In Iraq, 61% of pharmacists followed up warning alerts about SFM from the national pharmacovigilance agency, but 25.6% were not willing to report SFM to the agency (Al-Jumaili et al., 2021).

### Readiness to implement regulatory directives

To strengthen surveillance systems and technologies to detect SFM in the supply chain, the European Union and many countries have instituted regulatory directives. A survey of pharmacists was conducted in England to examine the readiness of the European Union's Falsified Medicines Directive (FMD) after its implementation and found that 39.2% reported ‘not at all’ and an additional 28.4% reported ‘not really’ (Barrett, 2020). There was substantial concern that the implementation of the directive could adversely affect business profitability and may not improve patient safety (Barrett, 2023). A similar study of pharmacists in Ireland found the majority of respondents perceived that the FMD requirements increased waiting times for patients (80.4%), reduced interaction time with patients (64.6%), and increased medicine shortages (34.6%) (Dalton et al., 2022). In Sweden, only 71% considered that the FMD could reduce the risk of SF drugs in the legal Swedish market (Persson et al., 2022). Interviews with pharmaceutical company professionals in EU countries show that their awareness of FMD was relatively high but insufficient to implement (Wlodarczak et al., 2017).

#### Perceived causes of SFM demand

It is often perceived that high demand with supply shortage and weak surveillance are the main drivers for the wide circulation and availability of SFMs (Fitsum et al., 2023). Pharmacists in high-income countries like Sweden also consider limited access to medicines, including very expensive nature of the cost and prescription requirement, to be the main motivation for people to purchase medicines from unauthorised online sources (Persson et al., 2024). A qualitative study of policymakers and community pharmacists in Khartoum, Sudan, suggests they believe that the unaffordable prices and nonavailability of the drugs are the major risk factors for consumers to get exposure to SFM (Alfadl et al., 2014). Weak knowledge of consumers about counterfeit drugs is also considered to increase their vulnerability. There is a concern that poor people are more vulnerable to SFM. A study in four sub-Saharan African countries (Ghana, Nigeria, Sierra Leone, and Uganda) suggests that less privileged populations are disproportionately exposed to SF medicines (Wagnild et al., 2024). Interviews of the general population in Sudan also confirmed that consumers may be motivated to purchase counterfeit drugs because of the high cost and non-accessibility of the drugs (Alfadl et al., 2012). A comparison of consumers between Sudan and Qatar suggests that consumers in lower economic settings have a more positive attitude toward buying CFM (Alfadl et al., 2016).

#### Online purchase of medicines and perceived risks

Online purchase increases people’s vulnerability to SFM exposure and often people lack knowledge and awareness associated with purchasing drugs from unauthorised sources. A survey of consumers in UAE suggests that less than 10% of the participants have purchased their medicines from online sources but most of these were non-prescription or over-the-counter drugs. About 78% of the respondents were not aware of the law of UAE regarding the purchase of medicines online, which is illegal (Ashames et al., 2019). A survey of patients in Hungary suggests that about 5% of the respondents purchased drugs online and only 7% of the online pharmacies required a prescription (Fittler et al., 2012). When ordering online drugs, only 85% of the test drugs were delivered and these drugs showed higher contamination and poor quality compared to authorised medications. High perceptions about the risk of purchasing drugs online have been suggested in some studies. About 82.8% of the respondents in a survey of hospital patients in Poland suggested that they were uncertain about the reliability of online pharmacies (Fittler et al., 2013). Another survey in Poland suggests that about 80% of respondents considered purchasing drugs on the Internet to be associated with a higher risk of receiving falsified drugs (Świeczkowski et al., 2021). Convenience was considered the most potential benefit of purchasing drugs through online pharmacies (Fittler et al., 2018). Uninsured individuals were more likely to import prescription drugs in the USA, likely through online purchases (Zullo et al., 2017). Covid-19 pandemic increased the propensity to purchase drugs online during the COVID-19 lockdown (Hamdan, 2023). In Nigeria, a survey of community pharmacists and consumers suggests that majority of the consumers expressed willingness to purchase medicines online and they expressed competitive pricing, confidentiality, convenience, and door-step services as the rationales for preferring online purchase (Ndem et al., 2019). An online survey conducted among predominately young participants from Amazon Mechanical Turk (MTurk) revealed a worrying perception, with these individuals rating online pharmacies as the safest option for purchasing medicines (Moureaud et al., 2021). Another survey with the MTurk sample suggests that consumers who were more knowledgeable about SFM had more favourable views on SFM than those who were not aware of the problem (Ofori-Parku & Park, 2022). In Jordan, about onethird of the participants believed that prescription drugs should be sold online, especially lifestyle medicines such as Viagra and birth control pills (Gharaibeh et al., 2023).

#### Interventions for improving knowledge and awareness to combat SFM

Only a few studies have focused on population-level interventions aimed at consumers and patients. One such study was conducted in Benin, which assessed the effectiveness of a public awareness campaign targeting the dangers of counterfeit medicines and the illicit medicine market in Cotonou (Abdoulaye et al., 2006). This survey evaluation revealed a significant increase in awareness among consumers, leading to a reduction in illicit medicine purchases. In a similar effort to improve people’s awareness, a Day of Action against counterfeit medications was organised in Brazzaville on March 22, 2018 (Degui et al., 2018). The event was considered successful, attracting over 1,500 visitors to the program. Furthermore, in Germany, a web-based interventional study targeted users of a simulated online pharmacy aimed to raise awareness of counterfeit medicine purchasing behaviours and test the influence of Internet interventions (Thomson et al., 2013). This study shows that internet interventions can significantly raise awareness about counterfeit medicines.

## Discussion

The escalating global challenge of SFM poses a significant threat to public health, straining healthcare systems and economies worldwide. SFM represents a pervasive problem that transcends international borders, affecting both high-income countries and LMICs. The emergence of online and internet pharmacies has further globalised this issue, complicating regulation and control efforts.

The health impacts of SF medications are profound and varied, leading to treatment failures, increased morbidity and mortality, and exacerbating the problem of drug resistance, especially with antibacterial and antimalarial medications. These issues extend beyond the immediate consequences for individuals, contributing to the global burden of disease and hindering public health progress, including jeopardising disease eradication campaigns and vaccination programs. Economically, the impact is significant, encompassing not only direct medical costs but also broader effects on earnings and productivity, thereby affecting both individual and public health efforts. However, a major challenge in tackling SF medicines is the difficulty in accurately capturing and reporting their impact. Much of the information about the mortality and morbidity associated with SF medicines is derived from sporadic reports and modeling, which highlights a gap in systematic surveillance and data collection. The current estimates derived from mathematical modeling are primarily available for a country or regional levels. No estimation is available at a global level. Although lifesaving medicines like anticancer medicines have been found as SFM in supply chains, no study has ever examined the health impact of such medicines on mortality or morbidity (Nistor et al., 2023; Venhuis et al., 2018).

One of the key challenges in addressing this issue is disentangling the effects of SF medicines from other contributing factors such as delayed treatment, incomplete medication course, and drug interactions. Accurately assessing the specific health impact of SF medicines requires a comprehensive approach that integrates surveillance, pharmacovigilance, and forensic analysis to systematically track and investigate suspected cases.

Enhancing pharmacovigilance systems to effectively monitor and analyze suspected cases of SF medicines is a critical step. Integrating SF medicine surveillance into existing pharmacovigilance frameworks would help in identifying patterns, quantifying impact, and enabling timely interventions. Additionally, healthcare professionals require targeted education and training to recognise and report suspected cases effectively.

A significant gap identified in our review is the lack of knowledge among healthcare providers and consumers regarding the risks and detection of SF medicines. To address this, incorporating SF medicine awareness into undergraduate curricula and continuous education for mid-career professionals – particularly for pharmacy, medical, and nursing students – could be an effective long-term strategy.

For instance, the WHO-FIP modules offer a structured framework for training healthcare professionals, providing them with the necessary competencies to identify, report, and mitigate the risks associated with SF medicines. Expanding the adoption of such standardised training programs globally could significantly strengthen the healthcare workforce’s ability to combat this issue.

The responsibilities of different stakeholders must also be clearly delineated. Regulators play a pivotal role in strengthening surveillance systems and enforcing stringent penalties for SF medicine distribution. Healthcare workers, including physicians, pharmacists, and nurses are critical in identifying and reporting suspected cases while educating patients on safe medication practices. Patients, on the other hand, require access to credible information and awareness campaigns to reduce their vulnerability to SF medicines.

LMICs are mostly affected but high-income countries are not immune, especially after the emergence of online medicines availability and purchase options (Ahmed et al., 2022; Lavorgna, 2015; Liang & Mackey, 2012; Liang & Mackey, 2012). The National Association of Boards of Pharmacy (NABP) reports that more than 95% of all online pharmacies are violating US public welfare and safety laws and mostly dispensing SF drugs (National Association of Boards of Pharmacy, 2019). A significant proportion of people purchase medicines online without being aware of the risks and dangers involved (Lavorgna, 2015). This lack of awareness contributes to the continued proliferation of these medicines, as patients may unknowingly purchase SF medicines, especially online. Healthcare providers, including pharmacists, are crucial in educating patients, but studies suggest they often lack the necessary training and confidence to effectively counsel patients on this issue.

Regulatory disparities between stringently and poorly regulated countries further complicate efforts to combat SF medicines. In high-income countries with robust pharmaceutical regulation and enforcement, SF medicine cases are often limited to online sales and isolated incidents. However, in LMICs, weak regulatory oversight, limited resources, and widespread informal markets allow SF medicines to proliferate more freely. Addressing this issue requires tailored interventions – such as regulatory capacity-building in weaker systems and targeted consumer education programs in settings where patients rely heavily on informal medicine vendors.

In terms of interventions and strategies, various measures have been implemented at both national and international levels to combat the issue of SF medicines. These measures include regulatory reforms, the adoption of technological solutions, and the execution of public awareness campaigns. However, the observed growth in the SFM market appears to be driven by both an actual increase in the prevalence of SF medicines and improved detection mechanisms. Advances in surveillance and reporting have led to more documented cases, though the scale of the issue remains difficult to quantify precisely. This situation underscores the need for more robust and multi-faceted approaches that not only address disruptions in the supply chain but also focus on educating both patients and healthcare providers about the dangers and realities of SF medicines. Our review suggests that healthcare providers and consumers, including patients, lack knowledge of risks, perceptions about vulnerability, and adequate competencies to identify SFM.

## Conclusion

While progress has been made, the increasing sophistication of those producing SF medicines, coupled with the challenges posed by globalisation and online sales, underscores the need for a coordinated, comprehensive approach. This includes strengthening regulatory frameworks, enhancing surveillance and reporting mechanisms, and improving public and provider awareness and education. A more proactive approach to data collection through international collaborations and systematic data-sharing platforms could further bridge the knowledge gap. Additionally, fostering collaborations between regulatory agencies, pharmaceutical companies, and academic institutions could enhance detection, reporting, and response efforts. It’s important to recognise that this issue can only be combated by addressing both aspects of the equation – supply as well as demand – with the understanding that interventions for each may be unique. By addressing these key areas, there is potential to make significant strides in the global fight against SFM, ensuring safer healthcare environments and better patient outcomes worldwide.

## Panel

### Definition of counterfeit medicine: standardisation challenges

Although the term ‘counterfeit drug’ has been used extensively in the literature, there is no consensus globally regarding its unified definition. The term has been interpreted from different perspectives, including patent and trademark infringement and intellectual property rights concerns. Counterfeit drugs are known by various names, including fake drugs, falsified drugs, and spurious drugs. Often, there is ambiguity in understanding the meaning of counterfeit medicines.

In 1992, the WHO first formally defined,

A counterfeit medicine is one which is deliberately and fraudulently mislabeled with respect to identity and/or source. Counterfeiting can apply to both branded and generic products and counterfeit products may include products with the correct ingredients, wrong ingredients, without active ingredients, with insufficient quantity of active ingredients or with fake packaging. (World Health Organization, 1992, 1999)

The issues of trademarks and intellectual property (IP) rights are reflected in the US federal government’s adoption of the definition of counterfeit drugs. The Food, Drug, and Cosmetic Act of the USA defines a counterfeit drug as

a drug which, or the container or labelling of which, without authorisation, bears the trademark, trade name, or other identifying mark, imprint, or device, or any likeness thereof, of a drug manufacturer, processor, packer, or distributor other than the person or persons who in fact manufactured, processed, packed, or distributed such drug and which thereby falsely purports or is represented to be the product of, or to have been packed or distributed by, such other drug manufacturer, processor, packer, or distributor. (21 U.S.C. 201(g)(2))

The European Union’s Falsified Medicines Directive (FMD) identifies counterfeit medical products as

Any medicinal product with a false representation of: (a) its identity, including its packaging, and labelling, its name or its composition as regards any of the ingredients including excipients and the strength of those ingredients; (b) its source, including its manufacturer, its country of manufacturing, its country of origin or its marketing authorisation holder; or (c) its history, including the records and documents relating to the distribution channels used [DIR 2001/83/EC Art 1(33)]. The definition does not include unintentional quality defects and is without prejudice to infringements of intellectual property rights.

The European Medicines Agency (EMA) defines counterfeit medicine as being ‘medicines that do not comply with intellectual-property rights or that infringe trademark law’ and falsified medicines as ‘fake medicines that are designed to mimic real medicines.’

Because of the concerns of trademarks and intellectual property (IP) rights issues, the WHO in 2011 adopted the term, substandard/spurious/falsely-labelled/falsified/counterfeit (SSFFC) medical products, and at the Seventieth World Health Assembly in 2017, the WHO adopted the term ‘Substandard and Falsified (SF) medical products.’

## Author contributions

SA conceived the study. EP and SA designed the search strategy, conducted the literature search, and identified the papers for inclusion. SA, EP and LM wrote the first draft of the manuscript. AK, RM, JCR, LK, HJM and PC revised the manuscripts and contributed to finalising the manuscript. All authors approved the final version of the manuscript.

## Supplementary Material

Supplemental Material
