# Supplementary material for: Assessing knowledge, attitudes, and practices and demand-side interventions for combating substandard and falsified medicines: a scoping review
Source: J Pharm Policy Pract. 2025 Sep 12;18(1):2550369. doi: 10.1080/20523211.2025.2550369 (PMC12434857; doi:10.1080/20523211.2025.2550369)
Supplement: Supplemental Material [file JPPP_A_2550369_SM1942.docx]

**Supplemental Material**

**Terminology Clarification**

In our review, we adopt “falsified” per the WHO 2017 definitions; however, we retain “counterfeit” in sections where the original studies consistently used that term.

**Table S1: Studies on adverse public health related outcomes**

| **Author and year** | **Country** | **Study Design** | **Study/Survey Population & Sample Size** | **Study Objectives** | **Key Findings** |
| --- | --- | --- | --- | --- | --- |
| Arens, van Wijk *et al.* 2016 | USA | Poisson control reporting system | 8 patients | Identify and describe the adverse effects associated with the ingestion of counterfeit alprazolam tablets containing fentanyl and etizolam | Central nervous system depression was prevalent among patients, with four experiencing cardiovascular issues that typically resolved within 24 hours. Lab analysis confirmed the presence of fentanyl, norfentanyl, and etizolam in patients and in a tablet, which contained 3.4 mg of fentanyl and 10.6 µg of etizolam |
| Beargie, Higgins *et al.* 2019 | Nigeria | Dynamic agent-based SAFARI model | Children under five with malaria in Nigeria | Assess health and economic impact of substandard and falsified (SF) antimalarials and evaluate potential interventions | Poor quality antimalarials cause 12,300 deaths and $892 million in costs annually. Interventions such as removing stock-outs, having only ACTs, and increasing care-seeking can save hundreds of millions annually |
| Evans, Higgins *et al*. 2019 | Uganda | Probabilistic agent-based SAFARI model | Children U5 | Assess the health equity impact of substandard and falsified antimalarials, focusing on socio-economic disparities and the urban/rural divide. | Patients bear 7.8% ($26.1MM) of the annual economic impact of malaria, with the majority affecting poor and rural communities, who experience higher mortality and out-of-pocket costs |
| Jackson, Higgins *et al.* 2020 | Zambia | Agent-based model, Substandard and Falsified Antimalarial Research Impact (SAFARI) simulation | Children under five in Zambia | To examine the health and economic impact of poor-quality antimalarials | Removing substandard and fake antimalarials could cut deaths in under-fives by 8.1% and prevent 937 hospitalizations, saving $8.5 million (MM) yearly |
| Kao et al. 2009 | Singapore | Clinical observational study | N=150 nondiabetic patients with severe hypoglycemia | To examine outbreak of severe hypoglycemia among non-diabetic patients | Four patients died. The cause was due to the contamination of illegal sexual-enhancement drugs with glyburide. |
| O'Donnell J, Tanz 2021 | USA | Analysis of data from CDC's State Unintentional Drug Overdose Reporting System | Overdose deaths from July 2019 to December 2021 in 29 states and DC, characteristics in 2021 in 34 states and DC | To describe trends in overdose deaths associated with counterfeit pill use and compare characteristics of deaths with and without evidence of counterfeit pill use | Counterfeit pill-related overdose deaths increased from 2% in late 2019 to 4.7% by 2021. There was a significant spike in the west from 4.7% to 14.7%. 41.4% involved illicit fentanyl, predominantly affecting younger, Hispanic or Latino individuals, often involving smoking as the consumption method. |
| O'Donnell J, Tanz LJ, Miller KD, *et al.* 2023 | USA | Secondary analysis of CDC's State Unintentional Drug Overdose Reporting System | Jurisdictions participating in CDC’s State Unintentional Drug Overdose Reporting System (SUDORS) | To assess quarterly percentages of overdose deaths with evidence of counterfeit pill. | A total of 106,293 overdose deaths occurred among 30 jurisdictions. The overall quarterly percentage of overdose deaths with evidence of counterfeit pill use increased from 2.0% during July–September 2019 to 4.7% during October–December 2021 |
| Ozawa S, Haynie DG., et al., 2019 | Democratic Republic of the Congo | Agent-based modeling | Children <5 years in Kinshasa province and Katanga region | Assess the health and economic impact of substandard and falsified antimalarials | Eliminating poor-quality antimalarials could reduce hospitalization costs by $5.38 million (63%) in Kinshasa and $19.2 million (62%)in Katanga |
| Ozawa, Evans *et al.* 2019 | Uganda | Probabilistic agent-based model (SAFARI) | Children under five with malaria in Uganda | Provide estimates of the health and economic impact of substandard and falsified (SF) antimalarials on children under five (U5) | The annual economic cost of malaria in children under five is $614MM, of which $31MM is due to SF antimalarials, contributing to $5.2MM in direct costs |
| Rahman et al, 2018 | Global | Summative analysis using data from published studies between 1972 and 2017 | Examined 1791 full-length articles in English and found 81 papers that qualitatively or quantitatively reported deaths and adverse health events due to SF drugs. |  | Only 48 studies reported adverse incidence and the authors calculated approximately 7200 causalities with 3604 deaths globally |
| Renschler JP, Walters 2015 | 39 sub-Saharan countries | Estimation using Latin hypercube sampling | Malaria-positive children under five consuming private sector antimalarials in 2013 | Estimate under-five deaths caused by ineffective treatment of malaria due to consumption of poor-quality antimalarials | Estimated 122,350 (IQR: 91,577-154,736) under-five deaths associated with poor-quality antimalarials, constituting 3.75% (IQR: 2.81-4.75%) of all under-five deaths in the 39 countries studied |
| Rentz et al., 2008 | Panama | Matched case-control study | 42 cases with and 140 control patients | Identify the cause of acute renal failure outbreak | Ingestion of cough syrup with contaminated diethyl glycol and glycerine was identified as the prime risk factor. |
| Tanz, Dinwiddie *et al.* 2022 | USA | Data analysis from the CDC's State Unintentional Drug Overdose Reporting System (SUDORS) | Adolescents aged 10-19 years | Describe the trends and characteristics of overdose deaths among adolescents from July 2019 to December 2021, focusing on opioids, particularly illicitly manufactured fentanyls (IMFs), and counterfeit pills | Adolescent overdose deaths doubled, with a 109% increase from July-December 2019 to July-December 2021. Deaths involving IMFs surged by 182%. Opioids were implicated in 90% of cases, IMFs in 83.9%, and counterfeit pills in nearly a quarter of the death |

**Table S2: Studies on knowledge, attitudes, and practice of healthcare providers and general public**

| **Author and year** | **Country** | **Study Design** | **Study/Survey Population & Sample Size** | | | **Study Objectives** | **Key Findings** |
| --- | --- | --- | --- | --- | --- | --- | --- |
| Abdoulaye I., Chastanier H. et al., 2006 | Benin | Survey evaluation of a public awareness campaign | Consumers in Cotonou, Benin | | | To assess the effectiveness of a public awareness campaign on the dangers of counterfeit medicines and illicit medicine market in Cotonou | Campaign effectively increased awareness and reduced illicit medicine purchases; most respondents preferred purchasing generics from public health facilities and pharmacies |
| Adigwe, OP., 2023 | Nigeria | Cross-sectional survey | 390 pharmacists, with 205 females (52.6%) and 185 males (47.4%) | | | Investigate Nigerian pharmacists' involvement in preventing and controlling counterfeit (CF) pharmaceuticals | 98.4% of pharmacists believe strict enforcement could reduce counterfeit drug sales. 68% see sourcing from reliable suppliers as their responsibility. About 65% cite weak enforcement as a key issue and emphasize the importance of educating patients about counterfeit drugs |
| Adigwe, OP., et al., 2022 | Nigeria | Cross-sectional survey | 390 Nigerian pharmacists | | | Assess the knowledge and practices of pharmacists concerning counterfeit medications (CFM) and explore the challenges in combating this issue | Major barriers included internet drug sales (72.68%), lack of inspections (90.93%), inadequate legislation (88.83%), poor collaboration (89.94%), and insufficient cross-border enforcement (90.43%). About 30.7% of pharmacists felt their knowledge and skills were inadequate for identifying counterfeit drugs |
| Alfadl, Ibrahim et al. 2012 | Sudan | Structured interview survey | 1003 participants in Sudan | | | To evaluate how attitudes, subjective norms, and motivations affect the intention to buy counterfeit drugs | Out of 11 hypotheses tested using structural equation modeling, only four were significantly confirmed, indicating that the expected factors influencing attitudes towards counterfeit drugs did not strongly affect purchase intentions, but the theory of planned behavior effectively explained the demand for counterfeit drugs |
| Alfadl, Ibrahim et al. 2013 | Sudan | Two-phase, quantitative survey | General public, with a pilot survey of 100 participants followed by a final survey of 1003 participants | | | To create and refine a "Consumer Behavior Toward Counterfeit Drugs Scale" from an initial 44 items to 41 items | The original scale of 44 items had a Cronbach's alpha of 0.818; refining it to 41 items improved the alpha to 0.862, indicating better internal consistency |
| Alfadl A.A., Ibrahim M.I.M, et al., 2014 | Sudan | Mixed-methods study | Policymakers, community pharmacists (qualitative); 1003 patients (structured survey) | | | Investigate how perceived unethical consumer behavior affects the decision to purchase counterfeit drugs in a developing country | Consumer awareness of societal impacts (t=0.575) and social stigma (β=-0.235, t=5.477) do not significantly influence counterfeit drug purchases |
| Alfadl, Ibrahim et al. 2016 | Sudan and Qatar | Structured face-to-face survey | 1,170 participants, including 1003 from Sudan and 167 from Qatar | | | To investigate consumer attitudes and motivations towards purchasing counterfeit products | Sudanese showed lower awareness of the societal consequences of buying counterfeit drugs, suggesting a more lenient stance on ethical purchasing, whereas wealthier Qatari participants demonstrated higher ethical considerations in their consumer behavior |
| Alfadl AA, Ibrahim MIM, et al., 2018 | Qatar | Cross-sectional study | General public and community pharmacists; 190 questionnaires distributed, 167 collected (87.8% response rate) | | | To assess perceptions and vulnerability to counterfeit medicines among the public and pharmacists | No significant perception difference; both groups showed low knowledge and moderate vulnerability to counterfeit medicines, with significant differences only in awareness, societal impact, price factors, and social influence on purchase intent (p ≤ 0.05) |
| Ali AB, Barrett R., 2023 | Saudi Arabia | Descriptive cross-sectional study | Community pharmacists in Saudi Arabia, surveyed electronically | | | To explore community pharmacists' knowledge regarding substandard and falsified medicines and their experiences with such products | 68.5% of pharmacists had no experience identifying (SF) medicines, and there was significant under-reporting |
| Al-Jumaili, Younus et al. 2021 | Iraq | Combined qualitative and quantitative methods | 587 pharmacists surveyed and 12 interviewed | | | Assess the effectiveness of alerts on substandard/falsified (S/F) medications from the Iraqi Pharmacovigilance Center (IqPhvc) to community pharmacies | Three-quarters recognized genuine medications by price, cost, and packaging; 72% of the participants had not received any training in the identification of S/F medications but 59.4% identified S/F medications using price stickers and national alerts |
| Ashames, Bhandare et al. 2019 | UAE | Cross-sectional study | 528 participants from the public | | | Assess the public perception and experience toward purchasing medicines from online sources | Under 10% of respondents purchased medicines online, predominantly nonprescription items (78%). The main motivations were lower prices and availability issues in local stores (both 43%). Despite these motivations, the overall sentiment towards online medicine purchases was generally negative |
| Barrett, R. 2020 | England | Cross-sectional study | Pharmacists in community pharmacies; N=501 pharmacies (random sampling, nationally representative sample) | | | Evaluated the readiness to implement the Falsified Medicines Directive (FMD) by community pharmacies in England. | Only 3.9% of surveyed pharmacists felt well-prepared; less than 3% reported cases of substandard medications; highlighted major gap in proactive action in underprivileged areas |
| Barrett and Al-Mousawi, 2018 | UK (Hampshire) | Postal survey | Pharmacies in Hampshire, 359 pharmacies surveyed, 50 responses (14% response rate) | | | To investigate pharmacists' perspectives regarding counterfeit and falsified medications (CFM) | Pharmacists consider counterfeit medications a significant threat to their profession (average rating of 4.02 on a five-point Likert scale). They believe training can improve their knowledge (average rating of 4.06) and their interventions could potentially halt the distribution of counterfeit medications to patients (average rating of 4.12) |
| Barrett R, 2022 | UK | Cross-sectional survey | 12,040 primary care pharmacies invited, 208 respondents | | | Assess FMD readiness and SFs prevalence vs. regulator detection | 61% ready for FMD, 54.8% expect improved safety, SFs prevalent in deprived areas. Bayesian simulation: 438 incidences (p = 0.030) with 3% SFs probability. Agency identified 15,238 SFs units (2019-2020). |
| Bashir, Galal et al. 2020 | Egypt | Cross-sectional study | 175 community pharmacists from Alexandria | | | Evaluate the awareness and practices towards counterfeit medicines among community pharmacists in Alexandria | Pharmacists aware of the seriousness of counterfeit medicines were significantly more likely to source from certified suppliers (P < 0.05), highlighting the importance of awareness in promoting safe procurement practices |
| Bautista K, Lee YA, et al.,2024 | Ghana | Case study using Decision-tree model | Vaginal and cesarean births data from Ghana's maternal health survey | | | To assess the health and economic impact of substandard uterotonics on postpartum hemorrhage (PPH) | Substandard uterotonics contribute to $18.8 million in economic burden, with significant out-of-pocket and productivity losses; improving uterotonic quality could prevent 20,000 PPH cases and save $6 million annuall |
| Binkowska-Bury, Januszewicz et al. 2013 | Poland | Questionnaire survey | | 1,788 primary healthcare providers and 377 lay respondents | Gain information concerning disparities in the understanding of the CFM phenomenon between healthcare workers and lay persons | | Medical professionals were less aware of the risks of purchasing drugs from non-pharmacy sources compared to the public, indicating a gap in understanding counterfeit medication risks and a lack of knowledge on reporting suspicious products and advising patients against unverified sources |
| Binkowska-Bury, Wolan et al. 2012 | Poland | Cross-sectional survey | | 201 healthcare professionals and 450 adult residents | Report on Polish hospital healthcare workers’ and lay persons’ knowledge about counterfeit medicine products | | Over 40% of the general public, 80% of nurses, and more than 90% of doctors were aware of the risks of counterfeit medicines from China or Ukraine. Despite high awareness, most healthcare professionals lacked knowledge on how to report suspicious medicines and often did not caution patients against purchasing drugs from unknown sources |
| Chaudhary S. 2023 | Nepal | Cross-sectional study | | 264 health professionals and pharmacists | Assess the awareness and attitude of health professionals and pharmacists towards counterfeit medicine | | Only 31.7% of participants had good knowledge of counterfeit medicine, with nurses, paramedics, and pharmacists scoring lower than doctors. However, 85.3% showed favorable attitudes towards combating counterfeit medicine |
| Dalton, Connery et al., 2022 | Ireland | Cross-sectional online survey | Community pharmacists, 618 valid responses out of 4,727 invited | | | To examine community pharmacists' views on the impact of the Falsified Medicines Directive (FMD) on their practice | 82% of pharmacists experienced longer patient wait times due to FMD regulations, 65% reported reduced patient interaction, and only 28% felt FMD enhanced patient safety, highlighting a discrepancy between regulatory intentions and practical outcomes in pharmacies |
| Ekoh, Chukwemeka et al. 2022 | Nigeria | Qualitative interviews | Purposefully selected group of 24 rural older adults in Southeast Nigeria | | | To explore the impact of the widespread counterfeit drug problem on rural older adults with chronic illnesses and how they navigate this challenge | A study highlights that many, especially women, buy counterfeit drugs from pharmacies and vendors, causing negative health and financial effects and pushing some towards alternative treatments, recommending stricter laws and better insurance for genuine medication access |
| El-Dahiyat, Fahelelbom et al., 2021 | Conducted online across Europe, Asia, Africa, America, and the Middle East | Online cross-sectional survey | Convenient sample of people aged 18 or older, with 320 participants | | | To determine the identification rate of substandard and falsified medications and its association with public knowledge | Only 30.6% of participants could accurately identify counterfeit drugs. Better identification was significantly associated with willingness to verify medication certification and report counterfeits to authorities, and among older, single, Asian, or American individuals |
| Fitsum Y., Werede A., et al.,2023 | Eritrea | Nationwide population-based cross-sectional survey conducted in December 2021 | 707 healthcare professionals from public and private health facilities | | | Assess the understanding, readiness, and response of Eritrean healthcare professionals regarding falsified medical products and identify intervention areas | Despite promising detection and reporting readiness among Eritrean healthcare professionals, significant knowledge gaps remain, with reporting barriers including a lack of awareness on reporting processes and delayed feedback from authorities |
| Fittler A., Lankó E., et al. 2012 | Hungary | Survey and quality assessment of online pharmacies and ordered medications | Over 500 patients; 163 online pharmacies | | | Estimate the prevalence of online drug purchases in Hungary, assess the quality of drugs sold online, and identify adequate methods for evaluating their safety | Nearly 5% of survey respondents purchased drugs online, with only 7% of pharmacies requiring a prescription; 85% of test-ordered medications were delivered, showing higher contamination and poor quality compared to authorized medications |
| Fittler, Lankó et al. 2013 | Hungary | Survey | 422 hospital patients | | | Assess the current situation of ordering medicines online and survey patient attitudes towards online drug purchases | Only 3.7% of patients consider buying medications or supplements online, with 8.4% having already made purchases. A significant 82.8% are uncertain about the reliability of online pharmacies |
| Fittler, Vida et al. 2018 | Hungary | Cross-sectional survey | 1055 Hungarian outpatients with a 77.23% response rate | | | Investigate the prevalence and perceptions of online medication purchases among Hungarian outpatients | Despite 82.65% of participants being aware they could purchase medications online, only 4.17% had done so, largely preferring retail pharmacies due to perceptions of benefits and risks associated with online purchases (P<.001) |
| Funestrand, Liu *et al.* 2019 | Sweden | Online survey | 200 physicians (100 emergency physicians and 100 general practitioners (Newton et al.)) | | | Assess EPs' and GPs' knowledge and experience with substandard and falsified (SF) medical products | In Sweden, 36.5% of surveyed physicians encountered suspected cases of patients using illegal and falsified medicines. 78.5% were familiar with the term SF, but there was a notable lack of knowledge on managing such patients and using the reporting system |
| Gharaibeh L, Alameri MA, Al-Kabariti AY, et al. 2023 | Jordan | Online survey | 428 adults 18 years or older reached through snowball distribution of questionnaire | | | Assess the extent of trust of the public in online drug purchasing | Public recognize the risks of purchasing drugs online but one-third favor the sale of prescription drugs online; 11.8% purchased drugs online and they have higher trust score. |
| Hamdan, 2023 | Jordan | Cross-sectional survey | 460 Internet-using adults over 18 in Jordan, selected via random sampling | | | To assess how COVID-19 lockdowns affected the prevalence of counterfeit drugs and medical supplies sold online | The survey indicated an increase in online medication purchases during COVID-19 lockdowns, linking the lockdown period to a rise in counterfeit drug and medical supply distribution in Jordan's online markets |
| Hertig, James et al. 2021 | USA | Survey | Pharmacists nationwide, 347 responses | | | To identify pharmacists' knowledge gaps concerning the risks posed by illegal online pharmacies | 58% of pharmacists were unsure how to advise on spotting illegal pharmacy websites, under 60% could visually judge a site's legitimacy, and 75% were unaware of tools to find trustworthy online pharmacies |
| Iloh GU, Akodu *et al.* 2021 | Nigeria | Survey | 178 Medical Practitioners (MP) in Abia State, Nigeria | | | Investigate Medical Practitioners' familiarity with and use of anti-counterfeit medicine technologies (ACMTs), and explore factors related to substandard and falsified medicines | Medical practitioners were universally aware of and used anti-counterfeit technologies, primarily labeling and mobile authentication. The main barrier was time constraints |
| Joda A, Amadi C, Adebayo O, *et al.* 2017 | Nigeria | Questionnaire survey | Healthcare providers in six local government areas of Lagos, Nigeria | | | Document healthcare providers' perceptions on the prevalence, affected drugs, implications, and control measures of drug counterfeiting | Respondents perceived a 41–50% counterfeiting rate in drugs, with anti-infectives, anti-malarials, and analgesics most affected. Key solutions included strengthening legislation and closing open drug markets |
| Khan MH, Akazawa M, Dararath E, et al. 2011 | Cambodia | Interviews using a semi-structured questionnaire | | Managing executives of 62 registered wholesalers of modern medicines in Cambodia (83.8% of the total wholesalers) | To investigate the awareness and practices of Cambodian medicine wholesalers concerning counterfeit medicines | | Approximately 13% of wholesalers encountered counterfeit medicines, with inconsistent definitions and perceptions of what constitutes a counterfeit |
| Kniazkov, Dube-Mwedzi et al. 2020 | Southern African Development Community (SADC) countries | Survey | | 16 National Medicines Regulatory Authorities (NMRAs) in the SADC region | To assess the existing frameworks and strategies within the SADC region for addressing substandard and falsified (SF) medical products | | Among the NMRAs that responded, 25% had policies including post-detection actions for SF products, 75% enforced regular product sampling, and 50% had established collaboration mechanisms to combat SF medical products |
| Kusynová Z, Kicken M, et al., 2021 | Global | Digital survey | 173 schools of pharmacy worldwide; 55 responses (32% response rate) | | | To gain insight into education on substandard and falsified (SF) medical products in pharmacy schools globally | 67% of schools teach about SF medical products, mainly focusing on detection and prevention; reporting is least taught (12%) |
| Kusynova, Bais et al. 2023 | Cameroon, Senegal, Tanzania | 20-question survey | 335 pharmacy students from three sub-Saharan universities | | | Determine if an educational course could enhance understanding of SF medical products | The educational course led to a 3.5-point average improvement in knowledge of SF medical products among students. Despite challenges like time and resource constraints, the course was well-received, highlighting the need for university support and early planning |
| Law E, Youmans S, 2010 | United States (California) | Survey | California pharmacists | | | To assess California pharmacists' knowledge and perceived barriers in addressing counterfeit medications | 59.3% of pharmacists view counterfeit drugs as a problem; key barriers include lack of resources (82.5%) and knowledge (46.8%) |
| Law E, Youmans SL 2011 | USA | Survey | California pharmacists | | | To examine California pharmacists' knowledge of counterfeit medications, the impact of technology, barriers to pharmacist involvement, and potential roles in combating counterfeit drugs | 59.3% of pharmacists see counterfeits as a professional issue; 46.8% reported lack of knowledge as a barrier, 82.5% cited lack of resources. Mixed views on the effectiveness of RFID technology |
| Lebed and Nemchenko 2021 | Ukraine | Comparative survey study | | State Service of Ukraine for Medicines and Drug Control officials, healthcare professionals, and consumers | Assess perceptions and identify consensus and differences on counterfeit drugs in Ukraine, aiming to develop prevention strategies | | Awareness of counterfeit medications varied among groups in Ukraine: 73.5% of consumers, 59.7% of State Medical Service experts, 42.3% of pharmacy specialists, and 11.4% of doctors were aware. Over 68% identified e-commerce as the main distribution channel, and about 70% supported stronger regulatory measures due to inadequate legislation |
| Lombardo, Marino et al. 2019 | Italy | Online survey | Community pharmacists, 668 responses | | | To evaluate community pharmacists' views on online pharmaceutical sales and their understanding of and experiences with falsified drugs | Only a small fraction of pharmacists supported online sales: 4.9% for prescription drugs, 25.4% for non-prescription drugs, and 51.6% for other products. Knowledge of falsified drugs was low: 24.5% unaware of active ingredient discrepancies, 46.4% oblivious to excipient differences, and 72.3% not recognizing the potential lethality of falsified drugs |
| Mhando L, Jande MB, Liwa A, Mwita S, et al. 2016 | Tanzania | Cross-sectional study | Public, interviewer-administered structured questionnaire and checklist | | | To assess public awareness and the ability to identify counterfeit antimalarial drugs based on appearance, packaging, labelling, and leaflets | 55.6% of respondents could distinguish between genuine and counterfeit antimalarial drugs |
| Moshoeshoe, Enslin et al. 2022 | South Africa | Analysis and in-depth interviews | | Government seizure reports from 2004-2017 and seven key stakeholders in medicine regulation and law enforcement | Evaluate the effectiveness of South Africa's frameworks in combating pharmaceutical counterfeiting | | The study highlighted inadequate legislation and enforcement challenges, with a significant lack of laws specifically targeting medicine counterfeiting. It emphasized the need for enhanced stakeholder engagement and information sharing |
| Moureaud, Hertig et al. 2021 | USA | Cross-sectional survey | 730 Amazon Mechanical Turk workers (from initial 1,002 responses) | | | Assess how fear and humor in public service announcements (PSAs) influence intentions to buy prescription drugs online and evaluate the impact of PSAs on purchase behaviors | Participants rated online pharmacies, Amazon, and Google+ as the most trusted platforms for purchasing medicines, while Kik and TikTok were seen as less safe. Over half reported purchasing narcotics (54.6%) and stimulants (52.0%) online. Higher education correlated with a lower likelihood of online medicine purchases, whereas being employed increased this likelihood |
| Nagaraj A, Tambi S, Biswas G, et al. 2015 | India | Cross-sectional questionnaire survey | | 300 participants: 100 medical practitioners, 100 dental practitioners, 100 medical storekeepers | Evaluate the knowledge, attitude, and practice towards counterfeit medication among doctors and medicine wholesale distributors in western India | | Medical practitioners had the highest knowledge scores; dental practitioners exhibited the most positive attitudes; best practices were most observed in medical storekeepers |
| Ndem E, Udoh A et al., 2019 | Nigeria, Uyo metropolis | Cross-sectional survey | | 60 community pharmacists, 500 consumers | Evaluate perceptions of online pharmacy services (OPS) | | The survey indicated that despite low awareness among consumers (28%) and pharmacists (57%), interest in online pharmacy services is high, with 67% of consumers and 92% of pharmacists seeing potential benefits. This interest is statistically linked to online shopping habits (P < 0.05), although concerns about counterfeit products and delivery reliability remain |
| Ofori-Parku and Park 2022 | USA | Survey | 427 U.S. consumers | | | Examine how consumer awareness, age, ethics, and social influences affect attitudes and intentions to buy counterfeit medicines | Knowledgeable consumers and those viewing counterfeit medicine consumption as ethical are more likely to view it favorably and intend to purchase. However, risk aversion reduces the likelihood of purchasing, and perceived benefits of counterfeit medicines do not significantly influence purchase intentions when considering risk perceptions and attitudes |
| Palamar JJ, Ciccarone D, et al., 2022 | United States | Retrospective analysis | National seizure data from High Intensity Drug Trafficking Areas (January 2018 – December 2021 | | | To assess trends in fentanyl seizures by form and quantity | Fentanyl-containing pill seizures increased nearly tenfold (from 68 to 635), and powder seizures quadrupled (from 424 to 1539). Pills as a proportion of total seizures doubled from 13.8% to 29.2% |
| Persson A., Troein M., et al. 2024 | Sweden | Qualitative analysis using interviews | 12 purposively selected pharmacists | | | Explore pharmacists' experience and knowledge about substandard and falsified (SF) medical products | Pharmacists recognize risk factors, protective measures, and areas for improvement regarding SF medical products, with their daily patient interactions offering opportunities to raise awareness and guide safer purchases |
| Persson, Troein et al., 2022 | Sweden | Digital survey | Employees at Sweden's top five pharmacy companies, potentially reaching 6200 staff; 4900 employees invited, 228 responses (5% response rate) | | | To assess the ability of community pharmacy staff to guide safer medication purchases, focusing on their knowledge and experiences with substandard and falsified (SF) medical products | Of the respondents, 89% were pharmacists, predominantly female (84%) and aged 35-49. While 80% were aware of SF medical products primarily through media, 74% did not recognize the European logo for authorized online pharmacies, highlighting a significant knowledge gap |
| Por C.S.,Keshavarzi, F., et al, 2020 | Malaysia | Descriptive cross-sectional study | General public, 387 participants | | | Assess public's knowledge, attitude, and practice (KAP) towards counterfeit and adulterated medicines  (CFAM); evaluate demographic influences on KAP; assess public opinion on education to combat CFAM | 43.9% of respondents had moderate knowledge and 54.5% had a positive attitude towards CFAM, 53% exhibited negative practices, with occupation, education, and employment status significantly influencing these factors.. |
| Rentz ED, Lewis L, et al., 2008 | Panama | Case-control study | 42 cases with acute renal failure; 140 matched controls | | | To investigate the cause and source of an outbreak causing acute renal failure and neurological dysfunction | Diethylene glycol (DEG)-contaminated prescription cough syrup was linked to illness (adjusted odds ratio: 31.0); 8% DEG contamination was found in syrup, 22% in glycerin. The outbreak led to a recall of ~60,000 bottles |
| Shahverdi S, Hajimiri M, et al, 2012 | Iran | Knowledge, attitude and practice (KAP) Study | 794 pharmacists at Iranian Pharmacist Association congress | | | Assess knowledge and measure professional attitude and practice of pharmacists about counterfeit drugs | Pharmacists showed low knowledge (mean correct practice responses: 13.62%) and practice concerning counterfeit drugs, with a positive attitude influenced by age and gender. Increasing weekly work hours negatively affected their proper practice |
| Sholy and Saliba 2018 | Lebanon | Cross-sectional study | Surveyed 849 Lebanese individuals | | | Assess Lebanese public awareness and attitudes towards CFM | 93.4% of respondents knew about counterfeit/falsified medicines, mainly from TV, but 28.9% couldn't reliably identify them |
| Sholy, Gard et al. 2018 | Lebanon | Survey using convenience sampling | 223 Lebanese pharmacists | | | Assess pharmacists' awareness and attitudes towards counterfeit medicines (CFM) | All pharmacists recognized CFMs, primarily through assessing medicine efficacy (67.7%) and price (66.8%). About 43% knew peers who dispensed CFMs, with 89.2% considering it unprofessional and 86.5% unethical, citing 'easy money' (87.9%) and high profit margins (86.5%) as reasons |
| Siraj, Gebre *et al.* 2022 | *Ethiopia* | Cross-sectional survey | 171 healthcare providers at Mizan-Tepi University Teaching Hospital | | | Evaluate the knowledge, attitudes, and practices regarding counterfeit medicines among healthcare providers | About 84% of participants were aware of counterfeit drugs; 15.8% recognized potential toxic impurities. Half could identify counterfeit drugs, and 61.4% supported stronger legal penalties |
| Sugita, Miyakawa et al. 2010 | Japan | Qualitative interviews | | Professionals from six corporations, including five pharmaceutical companies and one chemical company, and an anti-counterfeit specialist | Gather information on the genuine and counterfeit phosphodiesterase type 5 inhibitors (PDE5Is) market, including prices, quantities, and market dynamics of genuine, OTC, generic, and counterfeit PDE5Is | | Japan's counterfeit PDE5 inhibitor market is valued at 25,000 million yen, 2.5 times larger than the legitimate market. There are growing distributions of cheaper alternatives, though genuine products maintain higher value |
| Świeczkowski D, Zdanowski S, et al., 2021 | Poland | Observational cross-sectional study using computer-assisted telephone interviews | 1200 total respondents; 800 with cardiovascular diseases, 400 controls | | | To investigate patient perspectives on falsified medicines | In a study of 1200 respondents, 67.01% of the cardiovascular group and 65.25% of the control group trusted community pharmacies for non-falsified drugs (p < 0.01), while those with cardiovascular diseases or poor financial status showed lower knowledge about falsified medications (OR = 0.64 and OR = 0.58, respectively). |
| Tobias S, Shapiro AM, et al., 2021 | Canada (British Columbia) | Observational study | 139 counterfeit alprazolam samples (out of 10,814 total drug samples) | | | To analyze the contents of counterfeit alprazolam in the unregulated market | Only 23.7% of samples contained alprazolam; 72.1% tested positive for benzodiazepines via immunoassay, with confirmatory tests showing various psychoactive substances in most samples |
| Vallersnes OM, Lund C, et al., 2009 | Norway (Oslo) | Observational study | 44 cases of probable scopolamine poisoning | | | To document clinical features, diagnosis, and response to scopolamine poisoning epidemic from fake Rohypnol pills | Main symptoms included mydriasis, hallucinations, and coma; anticholinergic syndrome was only recognized post-forensic analysis, altering diagnosis and treatment approach |
| Wagiealla W. W., ShantierS. W., et al., 2022 | Sudan | Cross-sectional study | General public, 386 participants | | | Assess awareness and attitude toward counterfeit medicines (CFMs) | The study revealed that 58% of respondents in Sudan are aware of CFMs, mainly through social media, 73% consider CFMs of inferior quality, and 80% have a positive attitude towards combating CFMs |
| Wagnild J.M., Lee D *et al.*, 2023 | Tanzania and Indonesia | Pilot study | 309 healthcare professionals (2017), 254 surveyed at baseline | | | Assess utility of smartphone app for reporting SF medical products | Barriers: identification challenges, system frustrations, fear of repercussions. Pilot: 36 reports submitted, 8 SF products identified, all addressed. Positive feedback from HCPs and national medicine regulatory authority (NMRA). App partially addressed barriers, not all (e.g., concerns of repercussions) |
| Wagnild JM, Akhter N., et al., ,2024 | Ghana, Nigeria, Sierra Leone, and Uganda | Cross-sectional survey data analysis using multivariate regression and structural equation modeling | Over 1000 participants per country | | | Analyze the effects of an information gap versus structural constraints on public exposure to substandard and falsified (SF) medicines | Less privileged groups are disproportionately exposed to SF medicines, with education campaigns potentially reducing risky practices but only when combined with broader reforms in pharmaceutical supply chains and public health services |
| WŁodarczak U, Swieczkowski D et al., 2017 | European Economic Area | Cross-sectional survey | 1,496 pharmaceutical company professionals (January 2016 - June 2016), 17.37% response rate | | | Examine awareness of FMD implementation | 99 women (39.3%) and 153 men (60.7%) participated. 95.7% were aware of FMD. Pharmacists, pharmaceutical industry workers, and respondents with different professional profiles were more aware than doctors (p-values: 0.0063, 0.0014, 0.0114) |
| Yadav V, Budania N, Mondal A, *et al.* 2018 | India | Questionnaire-based, cross-sectional study | 100 registered doctors at SHKM Govt. Medical College, Nuh, Haryana | | | Assess the knowledge and exposure of doctors to counterfeit drugs and their perspectives on the impact on the health system | Only 57.8% (46/80) of doctors had accurate knowledge of counterfeit drugs, yet 90% (72/80) were aware of their dangerous effects. Over 50% believed modern technology can help control drug counterfeiting |
| Zullo AR, Howe CJ, et al., 2017 | United States | Cross-sectional study using data from the National Health Interview Survey (NHIS) from 2011-2013 | 87,494 individuals from the NHIS | | | To estimate the marginal association between lack of health insurance and the risk of personal prescription drug importation (PPDI) within various U.S. subpopulations | Uninsured individuals showed a higher prevalence of prescription drug importation. |
